# Food-derived bioactive oligopeptide iron complexes ameliorate iron deficiency anemia and offspring development in pregnant rats

**DOI:** 10.3389/fnut.2022.997006

**Published:** 2022-09-07

**Authors:** Wenfei Pan, He Gao, Xiaoling Ying, Caiju Xu, Xiang Ye, Yelin Shao, Mengdi Hua, Jie Shao, Xinxue Zhang, Shaowei Fu, Min Yang

**Affiliations:** ^1^Department of Nutrition and Food Hygiene School of Public Health, and Center of Clinical Big Data and Analytics of The Second Affiliated Hospital, Zhejiang University School of Medicine, Hangzhou, China; ^2^Zhejiang Provincial Centre for Disease Control and Prevention, Hangzhou, China; ^3^Department of Child Health Care, Children's Hospital, Zhejiang University School of Medicine, Hangzhou, China; ^4^National Clinical Research Center for Child Health, Hangzhou, China; ^5^Beijing Engineering Research Center of Protein and Functional Peptides, China National Research Institute of Food and Fermentation Industries Co., Ltd., Beijing, China

**Keywords:** food-derived bioactive oligopeptide iron complex, ferrous sulfate, iron deficiency anemia, pregnant rats, offspring development

## Abstract

This study aimed to investigate anemia treatment and other potential effects of two food-derived bioactive oligopeptide iron complexes on pregnant rats with iron deficiency anemia (IDA) and their offspring. Rats with IDA were established with a low iron diet and then mated. There were one control group and seven randomly assigned groups of pregnant rats with IDA: Control group [Control, 40 ppm ferrous sulfate (FeSO_4_)]; IDA model group (ID, 4 ppm FeSO_4_), three high-iron groups (H-FeSO_4_, 400 ppm FeSO_4_; MCOP-Fe, 400 ppm marine fish oligopeptide iron complex; WCOP-Fe, 400 ppm whey protein oligopeptide iron complex) and three low-iron groups (L-FeSO_4_, 40 ppm FeSO_4_; MOP-Fe, 40 ppm marine fish oligopeptide iron complex; WOP-Fe, 40 ppm whey protein oligopeptide iron complex). Rats in each group were fed the corresponding special diet during pregnancy until the day of delivery. After different doses of iron supplement, serum hemoglobin, iron, and ferritin levels in rats with IDA were significantly increased to normal levels (*P* < 0.05). Serum iron levels were significantly lower in two food-derived bioactive oligopeptide low-iron complex groups than in the low FeSO_4_ group (*P*<0.05). Liver malondialdehyde levels were significantly increased in the three high-iron groups compared with the other five groups (*P* < 0.05), and hemosiderin deposition was observed in liver tissue, indicating that the iron dose was overloaded and aggravated the peroxidative damage in pregnant rats. Liver inflammation was reduced in the three low-iron groups. Tumor necrosis factor α secretion was significantly decreased in all groups with supplemented oligopeptide (*P* < 0.05), with the concentration of tumor necrosis factor α declining to normal levels in the two whey protein oligopeptide iron complex groups. In the marine fish oligopeptide iron complex groups, body length, tail length, and weight of offspring were significantly increased (*P* < 0.05) and reached normal levels. Therefore, food-derived bioactive oligopeptide (derived from marine fish skin and milk) iron complexes may be an effective type of iron supplement for pregnancy to improve anemia, as well as reduce the side effects of iron overload, and improve the growth and nutritional status of offspring.

## Introduction

Anemia in pregnancy is a common global health problem that is gaining attention and interest in the clinical medicine and public health fields. The World Health Organization estimates that 36.5 [34.0–39.1]% of pregnant women worldwide (aged 15–49) are anemic (1, 2). Although some degree of dilutional anemia is part of normal physiology during gestation, maternal iron deficiency anemia (IDA) can lead to adverse pregnancy and newborn outcomes. These outcomes include severe maternal morbidity (3), as well as cognitive defects, stillbirth, low birth weight, and infant mortality (4–8). Physiological conditions of pregnancy can promote IDA, with the maximum absorption of iron from the diet less than the body's requirements for iron, resulting in the risk of iron deficiency (2). Inappropriate iron supplements may provoke a series of side effects (9, 10), and it remains challenging to find an optimal iron replacement product for the treatment of IDA.

Oral iron supplements provide a treatment for most individuals with iron deficiency, especially for those diagnosed with anemia in the first trimester. Ferrous sulfate (FeSO_4_) is a common oral iron treatment because it exhibits superior safety, and is inexpensive and widely available. However, oral iron supplements, especially traditional inorganic iron, may lead to iron overload (9, 10) and toxic effects, such as inflammation, oxidative stress, and intestinal damage (11–14). Therefore, new formulations have been developed (2). Polysaccharide iron complex or heme iron polypeptide have been investigated as treatments, but their effects were not ideal (15, 16). Food-derived bioactive peptides, as novel iron complex carriers, offer high bioavailability *in vivo* (17, 18) and have attracted broad research interest (19). These peptides can form a stable structure with iron, potentially decreasing the Fe pro-oxidant effect that is responsible for damage of the gastrointestinal mucosa (20). Furthermore, peptide-Fe complexes can exert an indirect antioxidant capacity and neutralize lipid radicals (21–24). However, data of these potential effects *in vivo* remains limited.

Although food-derived bioactive oligopeptide iron complexes, like β-lactoglobulin hydrolysate-iron complex and oat peptides-ferrous chelate, have been used as iron supplements in many studies, the effects on pregnancy and newborn are not clear. Therefore, we aimed to use the pregnant rat with IDA model to explore the effects of food-derived bioactive oligopeptide iron complexes (derived from marine fish skin and milk) on (1) iron deficiency anemia, (2) side effects caused by iron overload, and (3) offspring development.

## Materials and methods

### Materials

The AIN-93G improved (FeSO_4_ used instead of iron citrate) and intervention diets were prepared by Beijing Keao Xieli Feed Co., Ltd. The iron content was detected by Pony Testing International Group (report number GNADPGZA1S1021938). Hemoglobin (Hb), tissue iron, superoxide dismutase (SOD), malondialdehyde (MDA), reduced glutathione (GSH), and total protein commercial reagent kits were purchased from Nanjing Jiancheng Bioengineering Inst. (Nanjing, Jiangsu, China). A tumor necrosis factor α (TNF-α) ELISA kit was purchased from Shanghai Yuyan Biotechnology Co., Ltd.

### Preparation of food-derived bioactive oligopeptide iron complexes

Two food-derived bioactive oligopeptide iron complexes used in this study were prepared in the laboratory of the Beijing Municipal Functional Peptide Engineering Research Center. Oligopeptides were obtained from salmon skins and milk by two-step enzymatic hydrolysis: 3,000 U/g protein (Alcalase), pH 8.5, at 60°C for 2 h, then 2,500 U/g protein (papain), pH 7.0, at 60°C for 2 h. Subsequently, the hydrolysate was heated at 100°C for 10 min to inactivate the enzymes, and supernatant was obtained after cooling and centrifugation at 6,000 × g for 15 min. The peptide solution (<1 kDa) in the hydrolysate was collected with an ultra-filtration membrane (1 kDa), and oligopeptide dry powder was obtained by spray drying. To prepare oligopeptide iron complexes, 8 g oligopeptide dry powder was dissolved in 200 mL distilled water, and 2 g ascorbic acid was added to prevent oxidation of Fe^2+^. After the pH was adjusted to 5, 2 g FeCl_2_·4H_2_O was added and the sample incubated using a water bath at 50°C for 60 min. After cooling to room temperature, four times the volume of anhydrous ethanol was added. The solution was mixed then left to settle for 1 h. Finally, the precipitate was collected by extraction filtration, and stored after drying.

### Animals

Sprague Dawley female (50–60 g, newly weaned) and male SPF rats (250–300 g) were purchased from Shanghai SLAC Laboratory Animal Co., Ltd. [license no.: SCXK (Hu)2017-0005]c. The animal study was reviewed and approved by the Experimental Animal Ethics Committee of Zhejiang University Medical College (ZJU20200055). The rats were reared in cages at the Experimental Animal Center of Zhejiang University at a regulated temperature of 20–24°C, relative humidity of 50–60%, and under 12 h/12 h light/dark cycles. Before the formal experiment, female rats were adaptively fed with AIN-93G improved feed and double distilled water (in order to prevent the interference of minerals in the drinking water and better control the quality) for 3 days, while male rats were fed with normal feed and deionized water.

### Establishment of pregnant rat model of iron deficiency anemia

**Figure 1:**
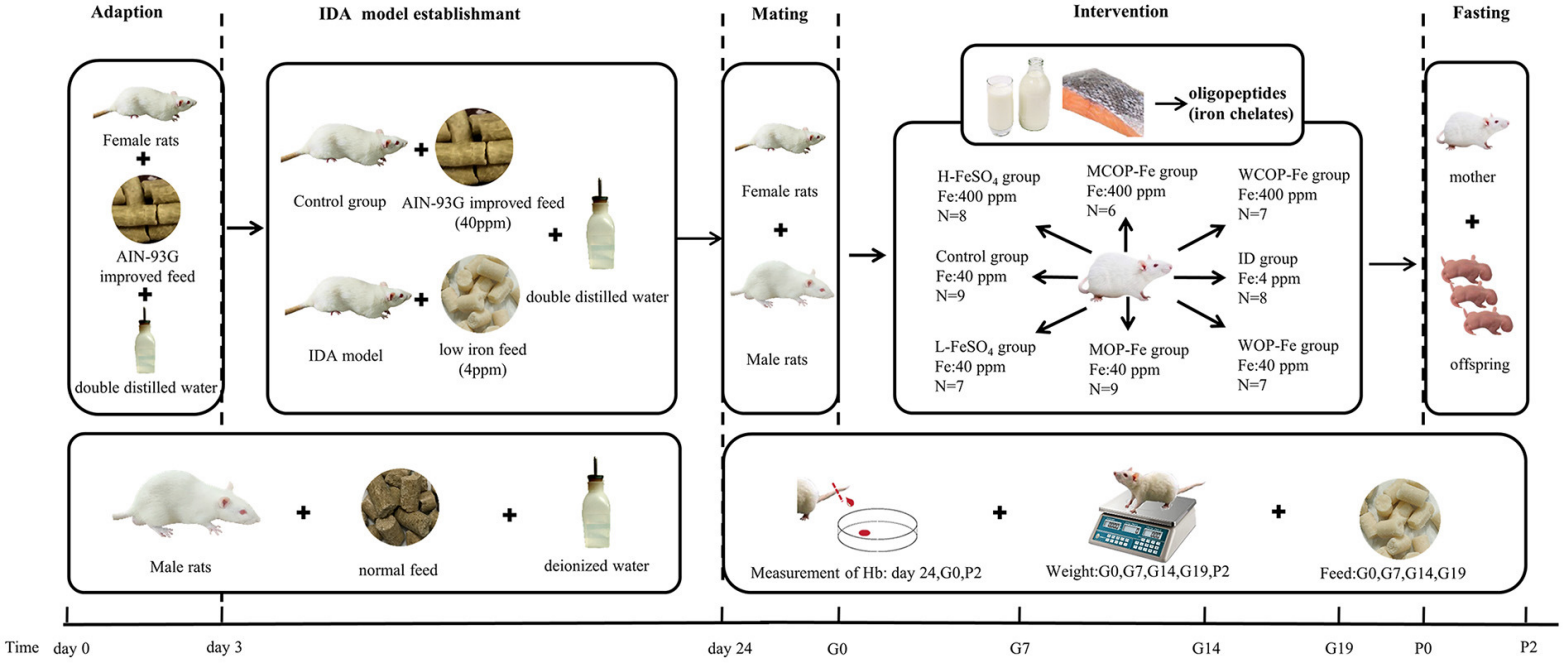
Scheme of IDA model establishment and iron intervention. IDA, iron deficiency anemia; Hb, Hemoglobin; ID, iron deficiency anemia model group (4 ppm FeSO_4_); H-FeSO_4_, 400 ppm FeSO_4_; L-FeSO_4_, 40 ppm FeSO_4_; MCOP-Fe, 400 ppm marine fish oligopeptide iron complex; MOP-Fe, 40 ppm marine fish oligopeptide iron complex; WCOP-Fe, 400 ppm whey protein oligopeptide iron complex; WOP-Fe, 40 ppm whey protein oligopeptide iron complex; G0, the day of pregnancy; G7, the Seventh day of pregnancy; G14, the 14th day of pregnancy; G19, the 19th day of pregnancy; P0, the day of delivery; P2, the second day after delivery.

The rat model of IDA and intervention during pregnancy are shown in Figure 1. Twenty-one female rats were purchased at one time, and after adaptive feeding, 2–3 rats were randomly selected as the Control group, which were fed the AIN-93G improved diet containing 40 ppm iron. The other rats were fed the low iron diet containing 4 ppm iron. On the 21st day of modeling, all rats were weighed and blood was collected from the tail vein to measure Hb. Rats with Hb <100 g/L were considered to be IDA (25), then remaining rats that did not meet the standard continued to be modeled. And in order to ensure that there was not much difference with body weight at the beginning of the intervention, we excluded rats that had still not been successfully modeled for more than 4 weeks.

After modeling, the normal and IDA female rats were caged with male rats 1:1, and day 0 of gestation was recorded when a vaginal plug was observed (26). Except the Control group, pregnant females were randomly divided into seven groups: IDA model group (ID, 4 ppm FeSO_4_), three high-iron groups (400 ppm FeSO_4_, H-FeSO_4_; 400 ppm marine fish oligopeptide iron complex, MCOP-Fe; 400 ppm whey protein oligopeptide iron complex, WCOP-Fe) and three low-iron groups (40 ppm FeSO_4_, L-FeSO_4_; 40 ppm marine fish oligopeptide iron complex, MOP-Fe; 40 ppm whey protein oligopeptide iron complex, WOP-Fe). Rats in each group were fed the corresponding special diet and double distilled water during pregnancy until the second day after delivery.

This operation was repeated until there were 6–9 females in each group.

### Sample collection

Rats were weighed and blood samples were collected from the tail vein to measure Hb at day 0, 7, 14, and 19 of gestation. After delivery, the female rats were fasted for 12 h and drank freely. Then, on the second day after delivery when the fasting was over, female rats were weighed and anesthetized by intraperitoneal injection of 2% sodium pentobarbital (0.25 mL/100g body weight), and the blink reflex was tested by cotton swab. After anesthesia, the incision was made along the abdominal midline, and blood samples were taken from the abdominal aorta of female rats. Then the plasma and serum samples were frozen at −80°C for further analysis after centrifugation. Heart, liver, spleen, kidney, and intestinal tissues were removed, rinsed with normal saline, weighed, and stored at −80°C for further analysis (27). In addition, male rats would be euthanized after all the mating was over.

The total number of fetuses, live fetuses, dead fetuses, and malformed fetuses were recorded. The body length (head and hip length), tail length, and weight of rat offspring at birth were recorded. All rat offspring were sacrificed and Hb content was measured.

### Analysis of hemoglobin and tissue iron

Hemoglobin levels and tissue iron levels of liver homogenates were measured with assay kits according to instructions of the manufacturer (Nanjing Jiancheng Bioengineering Institute, Nanjing, China).

### Analysis of serum biochemical indicators

Serum biochemistry analysis was performed on a fully automatic biochemical analyzer (7180, Hitachi, Japan). Measurements included serum iron (FE), ferritin (FER), transferrin (TRF), unsaturated iron-bonding capacity, and cell total iron-binding capacity levels.

### Analysis of oxidative stress biomarkers and TNF-α

Liver tissue was homogenized in normal saline to prepare 10% liver homogenate, and the supernatant was collected after centrifugation. The protein concentration, levels of MDA and GSH, and the activity of SOD in liver homogenates were measured with assay kits according to instructions of the manufacturer (28) (Nanjing Jiancheng Bioengineering Institute, Nanjing, China). The values were normalized to total protein content in liver tissue samples. Serum levels of TNF-α were measured by ELISA kits according to the manufacturer's protocols (Shanghai Yuyan Biotechnology Co., Ltd., Shanghai, China).

### Histomorphological analysis

At the time of sample collection, 10% formalin was used to fix the liver, duodenum, ileum, and colon. Tissue sections were then embedded in paraffin, stained with hematoxylin and eosin, and analyzed under light microscopy (29) ( ×400, ×200, and ×100 magnification, Laboratory of Pathology analysis, Zhejiang Provincial Center for Disease Control and Prevention).

### Statistical analysis

The data were expressed as mean ± standard deviation (SD). *T*-test and one-way ANOVA analyses were performed to compare the difference between two or multiple groups, and LSD analyses were used for comparison between groups. R 4.2.0 and GraphPad Prism 8 were used for statistical analysis and plotting. *P* < 0.05 was regarded as a significant level.

## Results

### Composition of two food-derived bioactive oligopeptide iron complexes

The compositions of two oligopeptide iron complexes are shown in Tables 1, 2. The protein content was above 70%, and the peptide content was above 50% of total weight. Total amino acids in marine fish oligopeptide iron complexes accounted for more than 80% of total weight, among which glycine was the highest, followed by glutamate, alanine, proline, arginine and aspartate. The total amino acids in whey protein oligopeptide iron complexes were more than 70% of total weight, among which the glutamate was the highest, followed by leucine, aspartate, lysine, proline, and isoleucine. According to the ideal protein conditions proposed by Food and Agriculture Organization of the United Nations/World Health Organization, the essential amino acids/total amino acids value of the protein should be about 40%, and the essential amino acids/non-essential amino acids value should be above 0.6. Amino acid composition detection showed that the whey protein oligopeptide iron complexes met the ideal protein conditions.

**Table 1:** Composition of food-derived bioactive oligopeptide iron complexes.

	**Protein content (wet base)**	**Peptide content (wet base)**	**Ash content (wet base)**	**Moisture content**
Detection method	GB5009.5-2016	GB/T22729	GB5009.4-2016	GB5009.3-2016
MCOP-Fe	94.81	78.47	3.66	2.87
MOP- Fe	91.73	77.25	2.05	5.85
WCOP-Fe	77.06	57.08	4.08	4.22
WOP-Fe	75.39	54.73	2.98	4.56

The unit is %. MCOP-Fe, 400 ppm marine fish oligopeptide iron complex; MOP-Fe, 40 ppm marine fish oligopeptide iron complex; WCOP-Fe, 400 ppm whey protein oligopeptide iron complex; WOP-Fe, 40 ppm whey protein oligopeptide iron complex.

**Table 2:** Amino acid composition of food-derived bioactive oligopeptide iron complexes.

	**MCOP-Fe**	**MOP-Fe**	**WCOP-Fe**	**WOP-Fe**
Amino acid (%)				
Asp	6.235	6.685	6.950	7.874
Thr	2.560	2.666	4.620	5.135
Ser	4.494	5.076	2.883	3.308
Glu	9.337	10.287	10.989	13.291
Gly	16.487	16.684	1.655	1.509
Ala	8.423	8.247	4.115	4.171
Val	2.691	2.767	4.222	4.452
(Cys)2	1.361	1.544	2.585	3.200
Met	2.265	2.399	1.724	1.861
Ile	1.873	1.905	4.708	5.046
Leu	3.627	3.719	7.708	8.246
Tyr	0.650	0.944	1.970	2.245
Phe	1.908	2.014	2.504	2.446
His	1.056	1.065	1.190	1.238
Lys	4.266	4.421	6.258	6.696
NH4	1.211	1.221	1.504	1.335
Arg	7.227	7.226	1.554	1.623
Pro	7.707	7.500	4.980	5.279
Total	83.378	86.370	72.119	78.955
Removal of NH4	82.167	85.149	70.615	77.620
E/T(%)	24.640	24.611	46.639	45.246
E/N	0.327	0.326	0.874	0.827

MCOP-Fe, 400 ppm marine fish oligopeptide iron complex; MOP-Fe, 40 ppm marine fish oligopeptide iron complex; WCOP-Fe, 400 ppm whey protein oligopeptide iron complex; WOP-Fe, 40 ppm whey protein oligopeptide iron complex; E/T, essential amino acids/total amino acids; E/N, essential amino acids/non-essential amino acids.

### Weights of pregnant rats during pregnancy and postpartum

As shown in Table 3, there were no significant differences in body weights between all groups on the day of pregnancy, or the second day after delivery (*P* > 0.05). However, on the 19th day of pregnancy, the weight of female rats in the ID group was significantly decreased compared with that in the Control group (*P* < 0.05), and after iron supplementation, the weight of female rats in all intervention groups returned to normal levels (*P* > 0.05).

**Table 3:** Weight of pregnant rats under iron deficiency and iron supplementation intervention (g).

**Group**	**N**	**G0**	**G19**	**P2**
Control	9	221.33 ± 20.22^a^	361.78 ± 22.19^ab^	268.67 ± 24.20^a^
ID	8	215.5 ± 18.31^a^	335.00 ± 17.50^c^	276.75 ± 12.89^a^
H- FeSO_4_	8	212.25 ± 14.63^a^	348.62 ± 18.65^bc^	275.25 ± 19.03^a^
L- FeSO_4_	7	205.43 ± 7.59^a^	361.43 ± 20.72^abc^	272.00 ± 18.10^a^
MCOP-Fe	6	219.00 ± 16.43^a^	380.00 ± 34.16^a^	285.83 ± 23.02^a^
MOP-Fe	9	209.33 ± 13.03^a^	368.89 ± 39.03^ab^	289.78 ± 23.85^a^
WCOP-Fe	7	222.57 ± 27.76^a^	377.71 ± 28.17^a^	288.00 ± 24.97^a^
WOP-Fe	7	208.00 ± 15.89^a^	363.29 ± 12.15^ab^	276.00 ± 16.34^a^
*P*		0.443	0.028	0.360

Values marked with different letters show significant differences. ID, iron deficiency anemia model group (4 ppm FeSO_4_); H-FeSO_4_, 400 ppm FeSO_4_; L-FeSO_4_, 40 ppm FeSO_4_; MCOP-Fe, 400 ppm marine fish oligopeptide iron complex; MOP-Fe, 40 ppm marine fish oligopeptide iron complex; WCOP-Fe, 400 ppm whey protein oligopeptide iron complex; WOP-Fe, 40 ppm whey protein oligopeptide iron complex.

### Hemoglobin levels in pregnant rats with IDA

**Figure 2:**
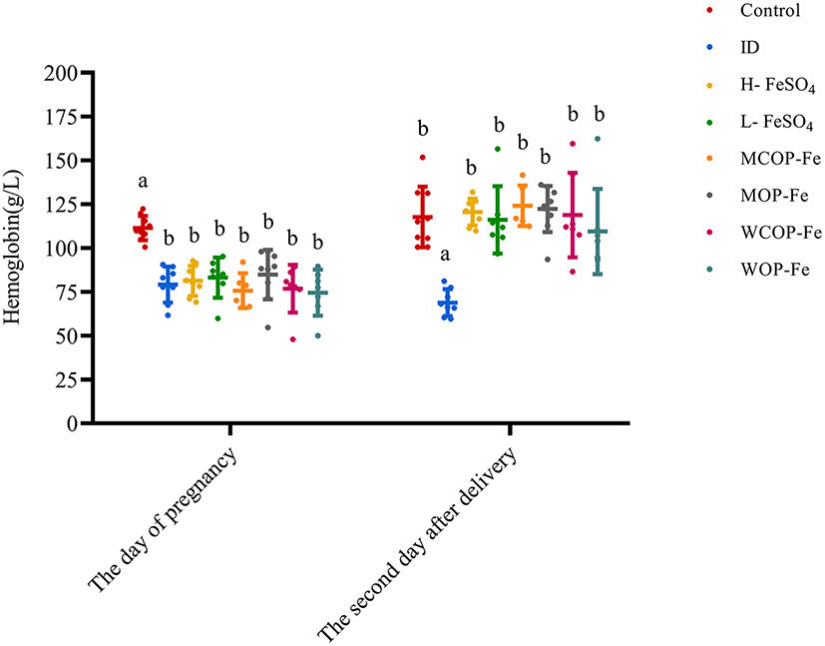
Hemoglobin in rats before and after intervention. Data are presented as mean ± SD, *P* < 0.05 is regarded as significant level, and values marked with different letters show significant differences. One-way ANOVA followed by LSD analyses was used for comparison among 8 different groups. ID, iron deficiency anemia model group (4 ppm FeSO_4_); H-FeSO_4_, 400 ppm FeSO_4_; L-FeSO_4_, 40 ppm FeSO_4_; MCOP-Fe, 400 ppm marine fish oligopeptide iron complex; MOP-Fe, 40 ppm marine fish oligopeptide iron complex; WCOP-Fe, 400 ppm whey protein oligopeptide iron complex; WOP-Fe, 40 ppm whey protein oligopeptide iron complex.

On day of pregnancy, except for the Control group, the Hb levels of pregnant rats in all groups were less than 100 g/L, and there were no statistical differences among the groups (*P* > 0.05), indicating that modeling was successful. On the second day after delivery, except for the ID group (*P* < 0.05), Hb levels in all iron intervention groups returned to normal (*P* > 0.05), suggesting that these intervention diets have similar effects on increasing Hb in rats during pregnancy (Figure 2).

### Organ index and iron-related indexes in pregnant rats with IDA

**Figure 3:**
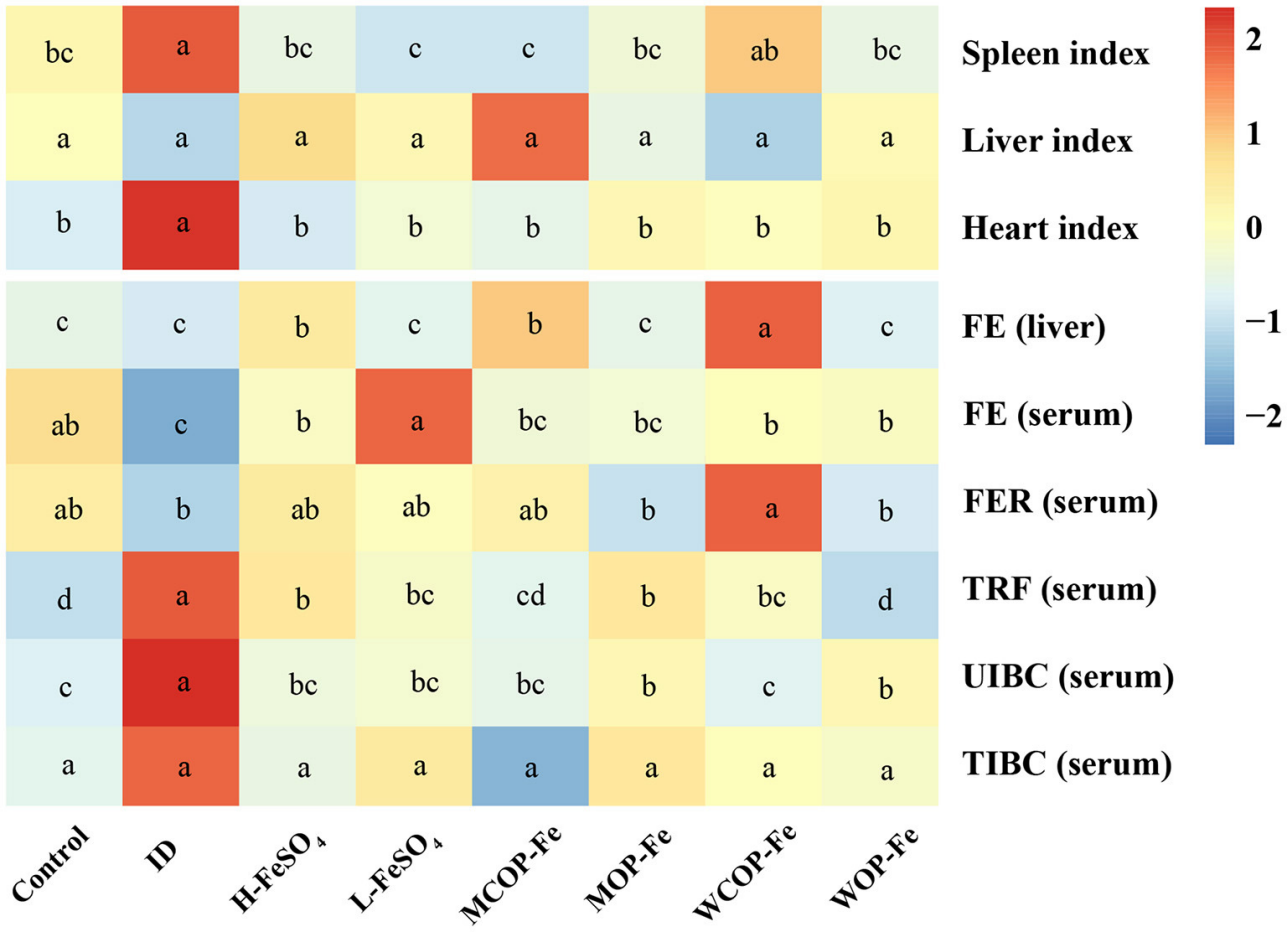
The organ index and iron related indexes of IDA pregnant rats after iron supplement treatment. The heatmap shows the relative content of the organ index and iron related indexes after normalization. The change in the intensity is marked by different colors, from blue to red as intensity enhancement. Values marked with different letters show significant differences. FE, Liver and serum iron; FER, serum ferritin; TRF, serum transferrin; UIBC, unsaturated iron binding ability; TIBC, total iron binding force; ID, iron deficiency anemia model group (4 ppm FeSO_4_); H-FeSO_4_, 400 ppm FeSO_4_; L-FeSO_4_, 40 ppm FeSO_4_; MCOP-Fe, 400 ppm marine fish oligopeptide iron complex; MOP-Fe, 40 ppm marine fish oligopeptide iron complex; WCOP-Fe, 400 ppm whey protein oligopeptide iron complex; WOP-Fe, 40 ppm whey protein oligopeptide iron complex.

The organ index and iron deficiency indexes of pregnant rats with IDA are illustrated in Figure 3. The results showed that the spleen and heart indexes were significantly increased in the ID group compared with the Control group (*P* < 0.05), while those of iron supplemented groups were normal. The iron content in liver tissues in all three high-iron groups was significantly higher than that in the other groups (*P* < 0.05). Serum iron reflects the availability of iron in the body and serum FER is the gold standard for iron storage in the body. In the current study, FE content was significantly lower in the ID group than that in the Control group (*P* < 0.05), and FER content tended to be lower, indicating that iron deficiency can consume iron storage in the body. After intervention with iron supplementation, FE in all intervention groups returned to normal levels, and FER levels also increased to varying degrees (all *P* > 0.05 compared with the Control group). Serum levels of TRF and unsaturated iron-binding ability were higher in the ID group than those in the Control group (*P* < 0.05), suggesting that iron deficiency affected iron circulation. After the intervention, TRF and unsaturated iron-binding capacity levels were decreased in all groups compared with the ID group (*P* < 0.05). Although FER levels showed no statistical difference in the three low-dose iron groups, FE was significantly lower in the MOP-Fe and WOP-Fe groups than in the L-FeSO_4_ group (*P* < 0.05), and levels of TRF were lowest in the WOP-Fe group (*P* < 0.05). These results suggested that these two food-derived bioactive oligopeptide iron complexes reduced free iron levels. Overall, the intervention prevented iron deficiency in the pregnant rats with IDA to some extent, and the oligopeptide iron complex was no less effective than FeSO_4_, even with high and low iron doses.

### Serum TNF-α and liver tissue oxidative stress biomarkers

**Figure 4:**
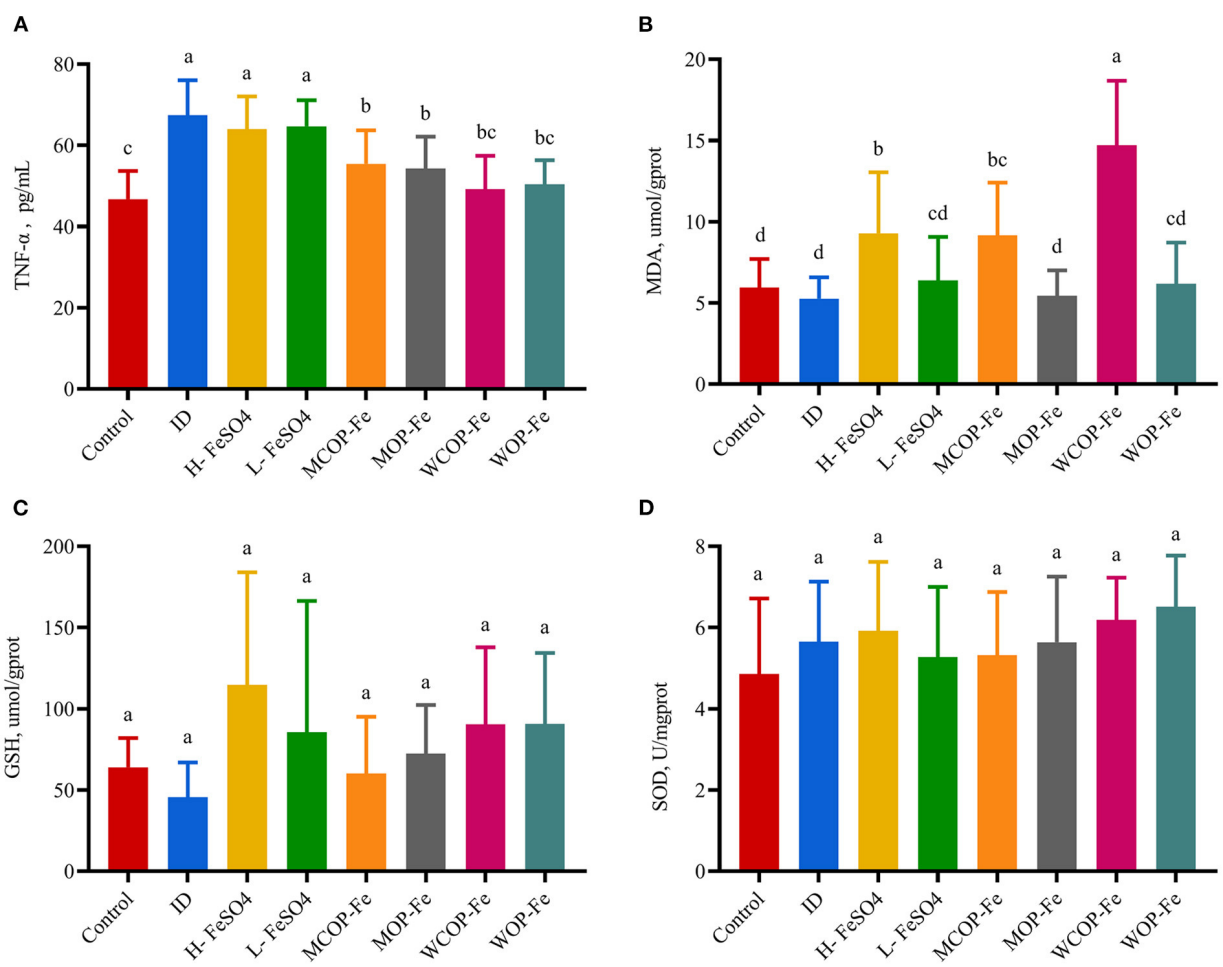
Effect on oxidative stress biomarkers in liver tissue and TNF-α in serum of IDA pregnant rats. Values marked with different letters show significant differences. **(A)** Effect on tumor necrosis factor α(TNF-α); **(B–D)** Effect on the malondialdehyde (MDA), geduced glutathione (GSH) and superoxide dismutase (SOD). ID, iron deficiency anemia model group (4 ppm FeSO_4_); H-FeSO_4_, 400 ppm FeSO_4_; L-FeSO_4_, 40 ppm FeSO_4_; MCOP-Fe, 400 ppm marine fish oligopeptide iron complex; MOP-Fe, 40 ppm marine fish oligopeptide iron complex; WCOP-Fe, 400 ppm whey protein oligopeptide iron complex; WOP-Fe, 40 ppm whey protein oligopeptide iron complex.

Serum TNF-α is a multifunctional cytokine that plays an important role in the inflammatory response. As shown in Figure 4, the concentration of TNF-α in the ID group was significantly increased (*P* < 0.05), indicating that iron deficiency caused an inflammatory response in the body. Supplementation with FeSO_4_ alone had no effect on TNF-α production, while two oligopeptide iron complexes significantly decreased TNF-α secretion (*P* < 0.05), with the WCOP-Fe and WOP-Fe groups exhibits concentrations of TNF-α restored to normal levels; showing that the whey protein oligopeptide iron complex may have a positive anti-inflammatory effect. In this experiment, MDA, GSH, and SOD were measured in liver homogenate to reflect the oxidative stress status of tissues. MDA is one of the most important products of membrane lipid peroxidation, which can aggravate membrane damage. GSH has antioxidant properties and helps maintain normal immune system function. And SOD, as an antioxidant metal enzyme, can reflect the ability of scavenging oxygen free radicals. In the current study, liver MDA levels were significantly increased in the three high-iron groups (*P* < 0.05) compared with the other five groups, suggesting that the high iron dose aggravated peroxidative damage in pregnant rats. No significant differences were found in liver GSH and SOD levels between all eight groups.

### Histological changes

**Figure 5:**
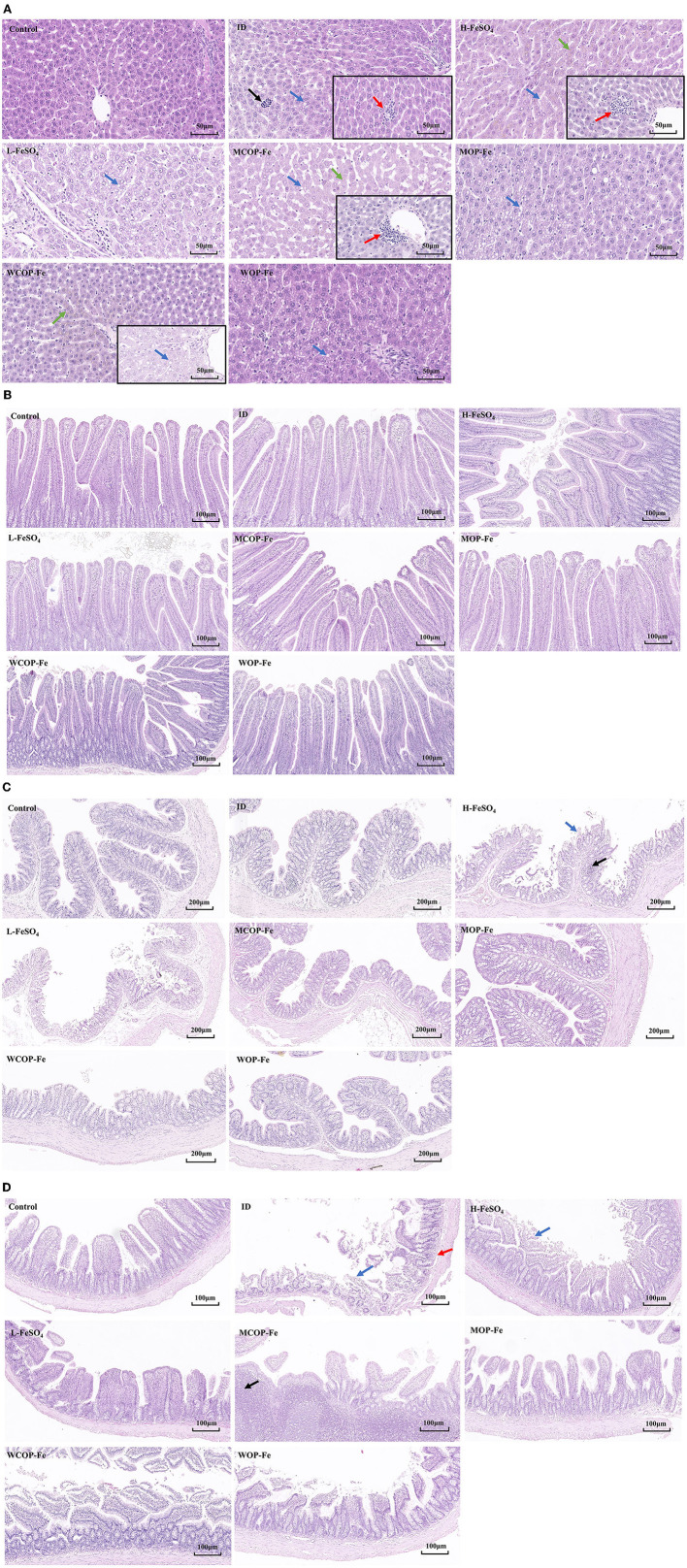
Hematoxylin-eosin staining results about liver and intestinal tissue sections of IDA pregnant rats. **(A)** Liver tissue sections of 8 groups. Black arrow, extramedullary hematopoietic; red arrow, infiltration of inflammatory cells; blue arrow, lipid drop of cavitation; green arrow, hemosiderin deposition. **(B)** Duodenum **(C)** Ileum **(D)** Colon tissue sections of 8 groups. Blue arrow, mucosal erosion; red arrow, lamina propria edema; black arrow, lymphoid hyperplasia. ID, iron deficiency anemia model group (4 ppm FeSO_4_); H-FeSO_4_, 400 ppm FeSO_4_; L-FeSO_4_, 40 ppm FeSO_4_; MCOP-Fe, 400 ppm marine fish oligopeptide iron complex; MOP-Fe, 40 ppm marine fish oligopeptide iron complex; WCOP-Fe, 400 ppm whey protein oligopeptide iron complex; WOP-Fe, 40 ppm whey protein oligopeptide iron complex.

The morphology of stained liver and intestinal tissue was observed under an optical microscope. There was the obvious presence of extramedullary hematopoiesis, fatty degeneration, and inflammatory reactions in liver tissue sections of the ID group. After the intervention, inflammatory responses were reduced in all three low-iron groups, although hemosiderin deposition was observed in all three high-iron groups (Supplementary Figure 1), suggesting possible iron overload. In addition, there were obvious inflammatory reactions in the H-FeSO_4_ and MCOP-Fe groups after intervention, which may be related to the side effects of iron overload. However, this phenomenon was not found in the WCOP-Fe group with the same iron content, indicating that it probably had anti-inflammatory properties (Figure 5A).

In the intestinal tissue, mucosal erosions, atrophy, a reduced number of intestinal glands, and edema of the mucosal lamina propria were observed in the ileum of the ID group, indicating that the low iron status mainly affected the ileal tissue (Figure 5C). After the intervention, the intestinal damage in all three low-iron groups was reduced, with the tissue structure basically normal. Similarly, the H-FeSO_4_ group had obvious colonic lymphoid follicular hyperplasia and mucosal erosion in the ileum as well as colon, and the MCOP-Fe group had ileal lymphoid follicular hyperplasia, while the intestinal tissue of WCOP-Fe group was generally normal (almost consistent with the liver tissue results) (Figures 5A–C).

### Pregnancy outcome and development of offspring at birth

**Figure 6:**
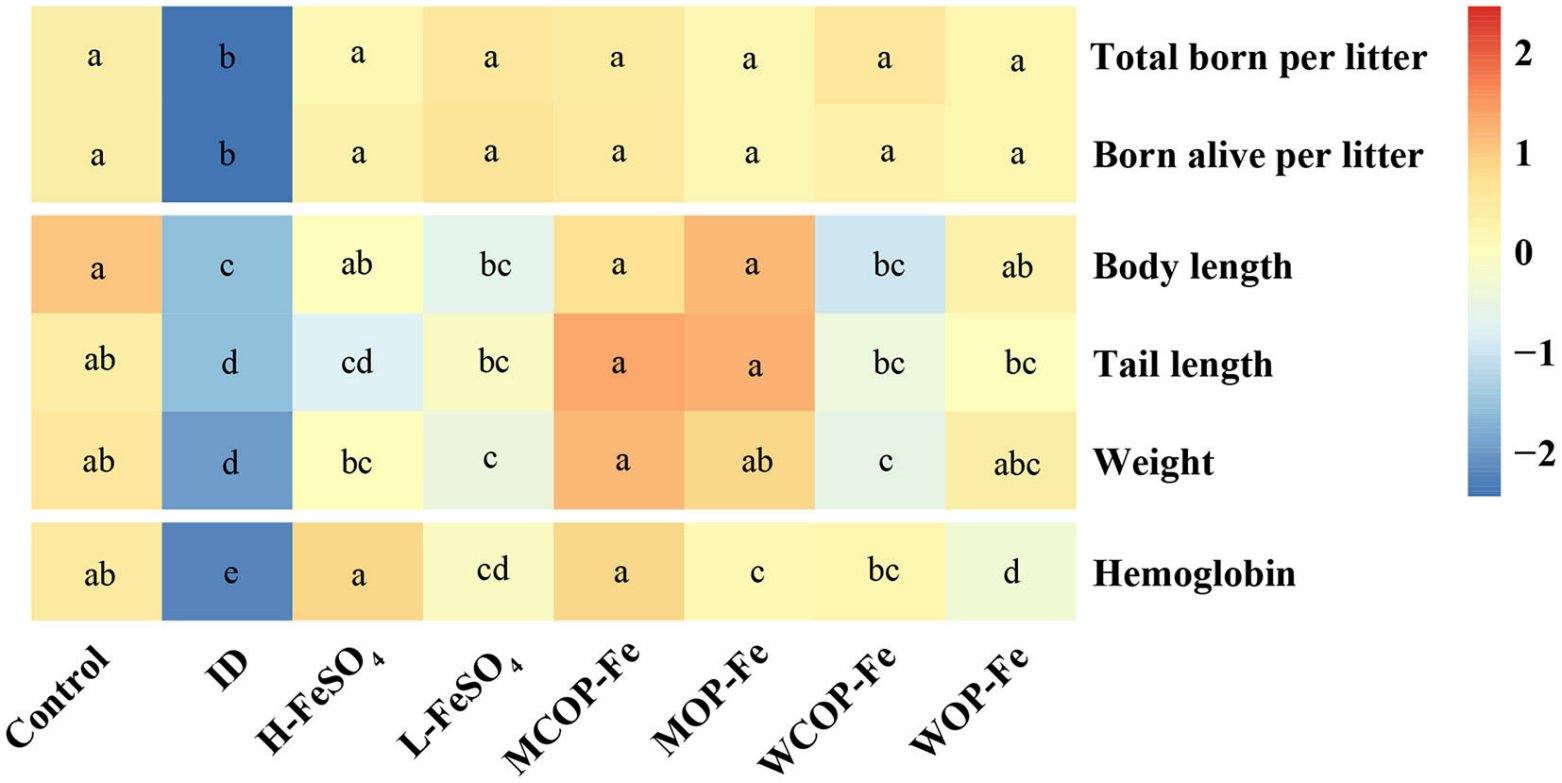
The pregnancy outcome and development of offspring at birth. The heatmap shows the pregnancy outcome and development of offspring at birth after normalization. Values marked with different letters show significant differences. ID, iron deficiency anemia model group (4 ppm FeSO_4_); H-FeSO_4_, 400 ppm FeSO_4_; L-FeSO_4_, 40 ppm FeSO_4_; MCOP-Fe, 400 ppm marine fish oligopeptide iron complex; MOP-Fe, 40 ppm marine fish oligopeptide iron complex; WCOP-Fe, 400 ppm whey protein oligopeptide iron complex; WOP-Fe, 40 ppm whey protein oligopeptide iron complex.

IDA caused the decrease of parturition rate, as shown in Table 4, the parturition rate of the ID group was 75%, which was higher after iron supplementation. The average number of total pups born per litter and total pups born alive per litter were significantly less in the ID group than those in the Control group (*P* < 0.05), and iron supplementation improved pregnancy outcomes. Body length, tail length, and weight of offspring were significantly decreased in the ID group compared with those in the Control group (*P* < 0.05), and the MCOP-Fe/MOP-Fe group showed a better recovery, suggesting that the marine fish oligopeptide iron complex may improve the growth of offspring during pregnancy in rats with IDA. Moreover, Hb levels of the ID group were decreased by nearly 50% compared with that of the Control group (*P* < 0.05), indicating that IDA in maternal rats had a great influence on offspring, and Hb in all the intervention groups was significantly increased (*P* < 0.05) (Figure 6).

**Table 4:** Pregnancy outcome of maternal rats.

	**Control**	**ID**	**H-FeSO_4_**	**L-FeSO_4_**	**MCOP-Fe**	**MOP-Fe**	**WCOP-Fe**	**WOP-Fe**
No. of pregnancy	9	8	8	7	6	9	7	7
No. of delivery	9	6	8	7	6	9	7	7
Parturition rate (%)	100	75	100	100	100	100	100	100

ID, iron deficiency anemia model group (4 ppm FeSO_4_); H-FeSO_4_, 400 ppm FeSO_4_; L-FeSO_4_, 40 ppm FeSO_4_; MCOP-Fe, 400 ppm marine fish oligopeptide iron complex; MOP-Fe, 40 ppm marine fish oligopeptide iron complex; WCOP-Fe, 400 ppm whey protein oligopeptide iron complex; WOP-Fe, 40 ppm whey protein oligopeptide iron complex.

## Discussion

Pregnant women are susceptible to iron deficiency and the resulting anemia, even in high-income countries (30). Iron supplements have been in practice for the past few decades, and food-derived iron fortification is a promising strategy for reducing the prevalence of anemia (31). This study used a stable pregnant rat model exhibiting IDA to explore the potential effects of two specially prepared food-derived bioactive oligopeptide iron complexes, in particular for the treatment of anemia.

Studies have shown that bioactive peptides obtained from different food sources have various biological functions, including antioxidant and immune regulation (32). However, evidence to date indicates that only a few active peptides are suitable for human consumption and available on the commercial market, with most of them derived from fish and milk proteins (33); such as the oligopeptides chosen for our experiment. Numerous food-derived peptides have the metal chelating ability to form complexes with divalent metals like calcium, zinc, and iron, and offer advantages of good bioavailability, high absorption, excellent stability, and high biosafety (33–35). In our experiment, Hb levels and iron-related indexes were significantly improved in all intervention groups compared with the ID group, with no significant differences observed in these parameters between the Control and treated groups. Two oligopeptide iron complexes had a good effect on reducing anemia and supplementing iron in pregnant rats with IDA, and these complexes were not inferior to the traditional iron supplement FeSO_4_. Similar to a previous study of pregnant rats, the effects of both high and low doses of complex iron on anemia in the current study were not significantly different from that of FeSO_4_ through a longer intervention (27).

Although no differences were found between the high-iron and low-iron groups in reducing anemia and iron-related indicators, the present study found that the liver iron content of the high-iron groups was significantly higher than the other groups, suggesting that this iron dose (400 ppm) may cause excessive deposition of liver iron. The liver is one of the most important tissues for iron storage, and excessive iron in the body is stored as ferritin and hemosiderin the in liver, spleen, bone marrow, and other tissues. The current study found hemosiderin in liver tissue sections of the three high-iron groups, consistent with previous work showing iron overload (36). Iron is an essential nutrient for cell metabolism, proliferation, and differentiation (37), however, excess iron deposited in tissues can cause damage (38). In our study, liver MDA levels were significantly increased in the three high-iron groups, indicating that iron overload may aggravate hepatic peroxidation damage in pregnant rats. Even though the two food-derived bioactive oligopeptide iron complexes were not very good at preventing oxidative stress from iron overload, there were some interesting findings. Among the three high-iron groups, group WCOP-Fe had normalized TNF-α levels and reduced inflammatory responses in liver and intestinal tissues, suggesting that the whey protein oligopeptide iron complex may have a protective effect against inflammatory reactions and intestinal damage caused by iron overload. The potential reason may also be the anti-inflammatory activity of whey protein oligopeptide itself. Previous studies have shown that whey protein, as well as bovine whey protein extract, can reduce inflammation (39, 40). In addition, the current whey protein oligopeptide was rich in leucine, which can reduce the expression of inflammatory genes in the liver, and also attenuate TNF-α-induced endothelial inflammation by normalizing the expression of TNF and endothelial nitric oxide synthase genes (41).

The presence of IDA not only affects pregnant women, but also the development of fetuses. Severe gestational iron deficiency can significantly increase the risk of low birth weight and growth restriction (42, 43), largely due to placental enlargement, junction enlargement, and altered gene expression in the placenta caused by maternal iron deficiency and anemia (44). The present study found that the low iron status of pregnant rats with IDA severely restricted the growth and development of offspring, making them severely anemic after birth, similar to the results of a previous study (27). However, the addition of marine fish oligopeptide, even at a low iron dose, can effectively restore the growth and development of offspring and reduce their anemic state. We speculate that these positive effects of marine fish oligopeptide used in the intervention may reflect the presence of arginine, which was about 4–5 times more abundant than that found in whey protein oligopeptide. Studies showed that supplementation of arginine nutrition can partially improve embryonic survival and development in mammals, including humans, pigs, sheep, and rats (45, 46). The specific underlying mechanism of arginine actions remains unclear, and to date, most of the biomedical applications of fish skin peptides focus on wound healing, skin anti-aging, inflammation reduction, and bone regeneration (47, 48). Therefore, the protective effect on fetuses may be an interesting and meaningful direction worthy of further exploration. Furthermore, marine fish oligopeptides are mostly derived from fish by-products, so effective utilization may greatly reduce discards (49).

However, there is also a limitation in our study. Strictly speaking, the design of our experiment is not completely independent, but more like a parallel experiment. One reason for this is the limitation of the site, as pregnant rats needed to be kept in a separate cage to prevent being startled (startled rats may end pregnancy or eat the baby). Another reason is animal ethics, male rats can be reused in parallel experiments to reduce the number of males rats. Therefore, we finally developed present research protocol used in our study.

In conclusion, IDA has adverse effects on pregnant rats as well as their offspring, However, excessive oral iron supplementation can also cause inflammation, oxidative stress damage, and intestinal tissue destruction. Food-derived bioactive oligopeptide (derived from marine fish skin and milk) iron complexes may be an effective prenatal iron supplement to reduce anemia and avoid some of the side effects of iron overload, and to improve the growth and nutritional status of offspring. Although the underlying mechanisms require further investigation, similar dietary oligopeptide iron supplements may be a promising option for iron supplementation.

## Data availability statement

The raw data supporting the conclusions of this article will be made available by the authors, without undue reservation.

## Ethics statement

The animal study was reviewed and approved by Experimental Animal Ethics Committee of Zhejiang University Medical College (ZJU20200055).

## Author contributions

MY contributed to conception and design of the study. WP and HG wrote the first draft of the manuscript. HG, WP, and XYi carried out specific experiments. WP performed the statistical analysis. All authors contributed to manuscript revision, read, and approved the submitted.

## Funding

This study was funded by Zhejiang Provincial Nature Science Foundation (LGF18H260003) and Beijing Municipal Functional Peptide Engineering Research Center Foundation (2018).

## Conflict of interest

Authors XZ and SF are employed by China National Research Institute of Food and Fermentation Industries Co., Ltd. The remaining authors declare that the research was conducted in the absence of any commercial or financial relationships that could be construed as a potential conflict of interest.

## Publisher's note

All claims expressed in this article are solely those of the authors and do not necessarily represent those of their affiliated organizations, or those of the publisher, the editors and the reviewers. Any product that may be evaluated in this article, or claim that may be made by its manufacturer, is not guaranteed or endorsed by the publisher.

## Abbreviations

IDA: iron deficiency anemia
FeSO_4_: ferrous sulfate
Hb: hemoglobin
SOD: superoxide dismutase
MDA: malondialdehyde
GSH: reduced glutathione
TNF-α: tumor necrosis factor α
ID: iron deficiency anemia model group (4 ppm FeSO_4_)
H-FeSO_4_: 400 ppm FeSO_4_
L-FeSO_4_: 40 ppm FeSO_4_
MCOP-Fe: 400 ppm marine fish oligopeptide iron complex
MOP-Fe: 40 ppm marine fish oligopeptide iron complex
WCOP-Fe: 400 ppm whey protein oligopeptide iron complex
WOP-Fe: 40 ppm whey protein oligopeptide iron complex
FE: serum iron
FER: ferritin
TRF: transferrin
SD: standard deviation.
