# Supplementary material for: Food-derived bioactive oligopeptide iron complexes ameliorate iron deficiency anemia and offspring development in pregnant rats
Source: Front Nutr. 2022 Sep 7;9:997006. doi: 10.3389/fnut.2022.997006 (PMC9490415; doi:10.3389/fnut.2022.997006)
Supplement: Supplementary file 1 [file Image_1.pdf]

Supplementary Material

1 Supplementary Figure

Supplementary Figure 1 | Prussian blue iron stain results about liver sections of IDA pregnant rats.

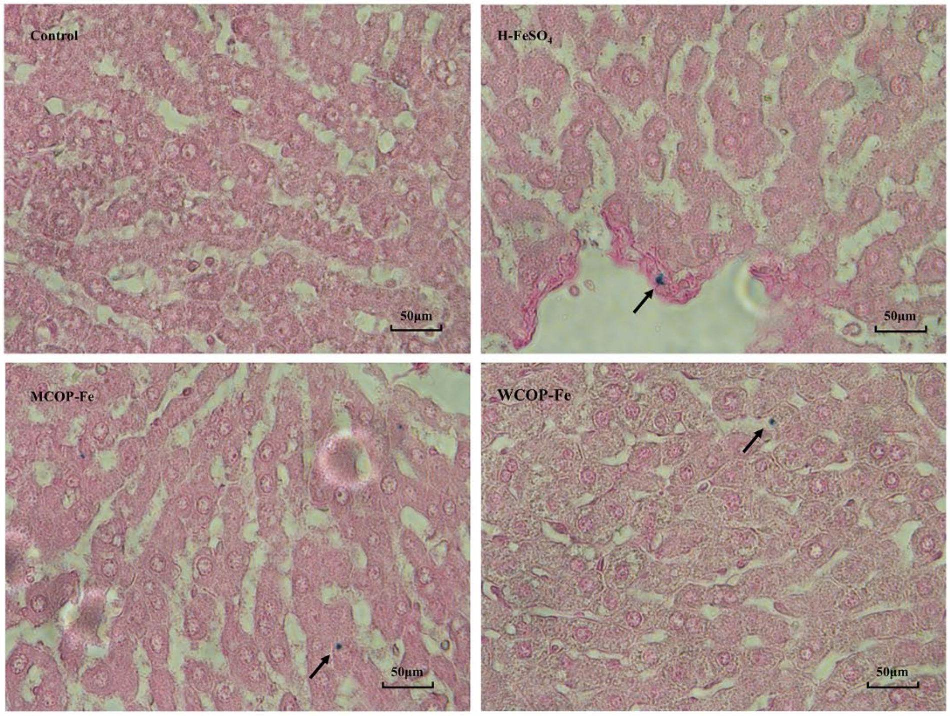

Liver tissue sections of the Control group and three high-iron groups. Black arrow, the blue granules of hemosiderin. H-$FeSO_4$, 400 ppm $FeSO_4$; MCOP-Fe, 400 ppm marine fish oligopeptide iron complex; WCOP-Fe, 400 ppm whey protein oligopeptide iron complex.
